# Galling-Free Dry Near-Net Forging of Titanium Using Massively Carbon-Supersaturated Tool Steel Dies

**DOI:** 10.3390/ma17194849

**Published:** 2024-10-01

**Authors:** Tatsuhiko Aizawa, Takeshi Kihara, Tomomi Shiratori

**Affiliations:** 1 Surface Engineering Design Laboratory, Shibaura Institute of Technology, Tokyo 144-0056, Japan; 2 Masunaga Optical Mfg. Co., Ltd., Fukui 918-8152, Japan; t.kihara@masunaga-opt.co.jp; 3 Faculty of Engineering, University of Toyama, Toyama 930-8555, Japan; shira@eng.u-toyama.ac.jp

**Keywords:** titanium, galling-free, dry, near-net forging, massively carbon supersaturation, tool steel dies, eyeglass frames, high product quality

## Abstract

Massively carbon-supersaturated (MCSed) tool steel dies were developed to make galling-free forging products from titanium bar feedstocks in dry conditions without lubricating oils. Two types of tool steel dies were used, SKD11 and ACD56, following the Japanese Industrial Standard (JIS). The plasma-immersion carburizing process was employed to induce massive carbon supersaturation in two kinds of tool steel dies at 673 K for 14.4 ks. A pure titanium bar was upset in a single stroke up to the reduction of thickness of 70% using the MCSed SKD11 die. Very few bulging displacements of the upset bar proved that μ = 0.05 on the contact surface of the MCSed SKD11 die to pure titanium work. Two continuous forging experiments were performed to demonstrate that an in situ lubrication mechanism played a role to prevent the contact surface from galling to titanium works in both laboratory- and industry-scaled forging processes. After precise microstructure analyses of the contact surface, the free-carbon film formed in situ acted as a lubricating tribofilm to reduce friction and adhesive wear in continuous forging processes. The MCSed ACD56 dies were also used to describe the galling-free forging behavior of manufacturing eyeglass frames and to evaluate the surface quality of the finished temples. The applied load was reduced by 30% when using the MCSed ACD56 dies. The average surface roughness of the forged product was also greatly reduced, from 4.12 μm to 0.99 μm, together with a reduction in roughness deviations. High qualification of forged products was preserved together with die life prolongation even in dry manufacturing conditions of the titanium and titanium alloys.

## 1. Introduction

The adhesion of work materials, or galling onto the die surfaces, is identified as one of the most essential issues in metal forming [1]. In particular, when forging in cold or stamping in hot the titanium and titanium alloys, there is a risk of severe galling since the fresh surfaces of the work are directly in contact with the die surface [2]. As is pointed out in [3,4], this galling is not suppressed even by solid lubrication or ceramic coatings. Hence, in industries, titanium and titanium alloy eyeglass frames and surgery tools have been fabricated in dry and cold conditions by multi-step forging processes, including the polishing and surface treatment steps to remove titanium oxide debris and fresh titanium adherents to die surfaces [5]. In the surface treatment substeps, the anodizing process is utilized to protect work materials from galling [6], and the oxide layers easily delaminate by themselves during the forging steps. Various coating methods and solid lubrication also have not provided a solution to save this long-step production procedure with high labor costs [7,8]. An innovative change is necessary to protect the die surface from galling even in dry, near-net forging processes [9].

The massive carbon supersaturation (MCS) process was proposed to impinge and store carbon interstitial atoms in die materials using low-temperature plasma immersion carburizing [10]. Titanium and β-phase titanium alloy bar feedstocks were reduced to a thickness of 70% with a low friction coefficient without galling and free-forged into complex-shaped beams [11]. In addition, a thick titanium plate was punched into gears with high dimensional accuracy and fully burnished gear surfaces under MQL (Minimum Quantity Lubrication) [12]. Furthermore, tiny titanium parts of eyeglass frames were successfully near-net forged in dry and cold conditions by reducing the manufacturing steps [13,14].

**Figure 1:**
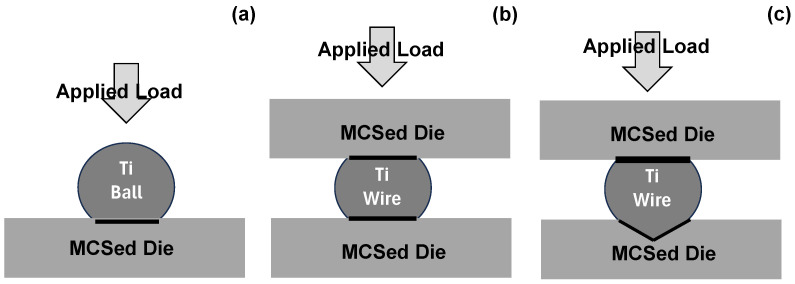
In situ formation of the free-carbon tribofilm onto the contact interface hot spots of MCSed tool steel dies in titanium work materials. (**a**) Tribofilm formation on the wear track during BOD testing. (**b**) Tribofilm formation on the MCSed die and titanium wire during upsetting. (**c**) Tribofilm formation on the expanding contact surfaces during near-net forging.

In the present paper, plasma-immersion carburizing is employed to make massice carbon supersaturation (MCS) treatment of SKD11 and ACD56 tool steel dies to demonstrate the galling-free forging of pure titanium bar feedstocks experimentally. Ball-on-disc (BOD) testing and two types of upsetting processes, at laboratory and industrial scales, were performed to analyze the contact surface conditions between the MCSed SKD11 die and the titanium work and to describe the in situ lubrication effect on the low friction and adhesive wear of the contact surface. As illustrated in Figure 1, the free-carbon tribofilm is formed on the contact surface in every case, and the incubation time is observed for nucleation and growth of the tribofilm to cover the hot spots on the contact surface. This in situ lubrication by tribofilm formation is characterized by a lower friction coefficient than 0.1 in dry conditions, e.g., μ = 0.07 in BOD testing and μ = 0.05 in upsetting. No essential difference in this lubrication process is recognized between the two upsetting processes at the laboratory and industry scales. The MCSed ACD56 dies are also used to allow for the dry near-net forging of titanium alloy bars in the manufacturing of eyeglass frames. The applied load is reduced by 30% because of a low level of friction on the contact surface, and the average surface roughness of the finished temples is significantly improved from 4.19 μm to 0.99 μm. This nontraditional die technology plays a significant role in precisely manufacturing titanium medical tools.

## 2. Materials and Methods

The plasma-immersion carburizing system (SKY005; YS-Electric Industry, Co., Ltd., Kofu, Japan) was utilized for massive carbon supersaturation at a low temperature. BOD testing was also used to measure variations in the friction coefficient with an increasing rotational-sliding distance. Both the upsetting and near-net forging processes are explained, together with a measurement of the applied load and product surface roughness.

### 2.1. Carbon Supersaturation Process

**Figure 2:**
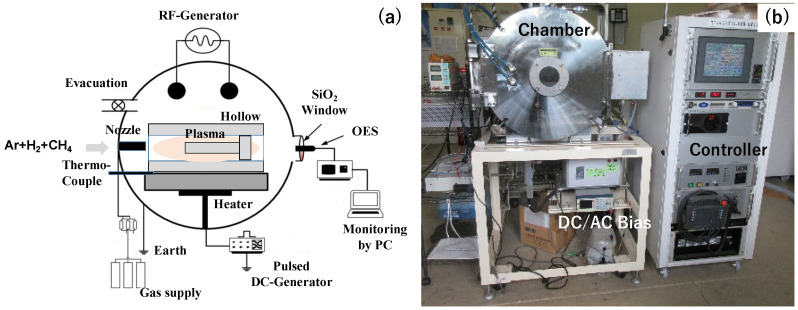
Plasma nitriding immersion system for massive nitrogen supersaturation into tool steels. (**a**) A schematic view of the whole system and (**b**) an overview of the whole system.

RF (Radio Frequency) and DC (Direct Current) plasmas were ignited and controlled to induce a nitrogen–hydrogen–methane plasma sheath onto the dies inside the hollow cathode in Figure 2a, as explained in [15]. The carbon ions and CH radicals were activated and densified in this hollow to enhance the carbon supersaturation process. The thermocouple was embedded into the base plate to monitor the process temperature. Through the silica window, the optical emission from plasmas was detected and analyzed by OES (Optical Emission Spectroscopy, Impedance, Co., Ltd., Dublin, Ireland). The whole system in Figure 2b consisted of the chamber, the DC/AC bias, the evacuation units, the gas supply units for argon, hydrogen, and methane gases, and the digital controlling system with a touch panel.

**Figure 3:**
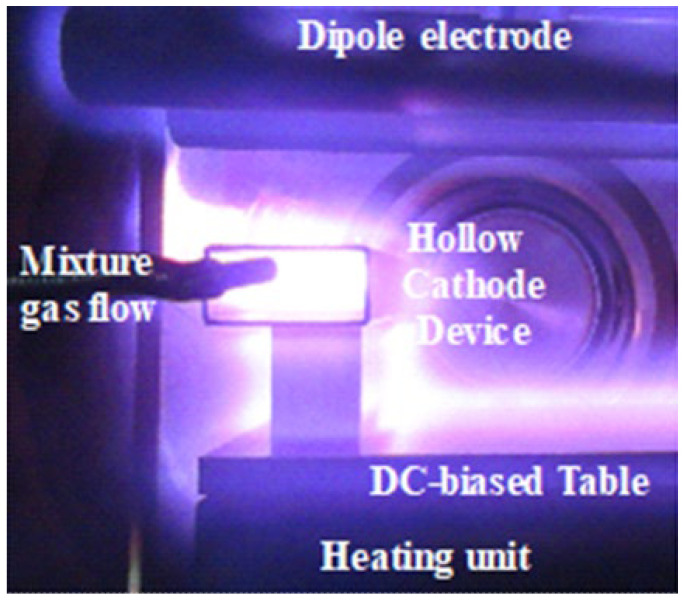
Nitrogen–hydrogen–methane plasma sheath surrounding the whole die specimen.

Figure 3 depicts an ignited nitrogen–hydrogen–methane plasma immersion in the hollow. After OES, both the carbon ions and CH radicals are highly densified in the plasmas to drive the carbon supersaturation to tool steel dies [16]. The densified population of these nuclei in the low-temperature plasma immersion is different from conventional plasma carburizing for the synthesis of carbide precipitates into steel dies [17]. In the following experiments, the above setup was placed into the chamber before evacuation to the base pressure of 0.01 Pa. The setup was heated under a pure argon atmosphere up to 673 K. After presputtering in the argon–hydrogen atmosphere by 50 Pa for 1.8 ks, methane gas was introduced as a carbon source. The carburizing was performed at 673 K for 14.4 ks under the mixture gas of Ar, H_2_, and CH_4_ by 50 Pa with a flow rate of 160 mL/min for argon, 20 mL/min for hydrogen, and 20 mL/min for methane. After carburizing, the setup was cooled in the nitrogen gas flow to 473 K.

### 2.2. BOD Testing

A tribotesting system (Tribometer, CSM; Bern, Switzerland) in the rotational sliding mode was utilized to measure the friction coefficient on the contact surface of the MCSed SKD11 substrate to a pure titanium ball with a diameter of 6.0 mm. The applied load (W) was 10 N, and the rotating speed (v) was 0.1 m/s. The frictional force (F) was measured up to a sliding distance of 500 m. The friction coefficient (μ) was defined by μ = F/W. 

### 2.3. Upsetting Process

A CNC (Computer Numerical Control)-stamper (MU-1; Precise Forming Laboratory, LLC., Tokyo, Japan) was utilized for an upsetting experiment on the laboratory scale. An MCSed type-SKD11 punch and die were respectively fixed into the upper and lower cassette die sets. The two die sets were cemented to the upper and lower bolsters of the CNC-stamping system with stroke control capability. The stroke was controlled by moving down and up the upper bolster to the specified position to reduce the thickness of the work materials. The loading speed was constant at 10 mm/s. A knuckle-joint stamper (AIDA160; AIDA, Co., Ltd.; Kanagawa, Japan) was utilized for the upsetting experiment with a maximum applied load of 1200 kN. SKD11 dies with and without MCS were used in this upsetting on the industry scale. 

### 2.4. Near-Net Forging Process

Type ACD56 tool steel with the hardness of HRC57 was prepared as a die for near-net forging with and without MCS. The knuckle-joint stamper was also used to shape the final product from its preform. There is a risk of severe galling in this forging process where the fresh titanium work directly contacts the die surface to roughen the product surface quality.

### 2.5. Material Characterization

SEM (Scanning Electron Microscopy; JEOL, Tokyo, Japan)–EDX (Electron Dispersive X-ray spectroscopy; JEOL, Tokyo, Japan) was utilized to analyze the contact surface between the die and work materials. Raman spectroscopy (NRS-7100; Nihonbunkou, Co., Ltd., Tokyo, Japan) was also used to describe the carbon structure on the contact surface.

## 3. Results

BOD testing was first performed to describe the in situ formation of the free-carbon tribofilm on the contact surface of the MCSed SD11 substrate against the titanium ball. The pure titanium bars were upset on the laboratory and industry scales using the MCSed punch and MCSed die, respectively. The frictional behavior on the contact surface of the MCSed dies to work, was analyzed by inverse analysis with the use of finite element analysis. Microstructure analysis was also performed to describe the in situ-formed tribofilm during upsetting. The near-net forging on the industry scale was also performed to describe the variation in the applied load and product height with an increasing number of strokes in the continuous forging process. The product quality was also evaluated by analyzing the surface roughness of the products.

### 3.1. In Situ Lubrication during BOD Testing

**Figure 4:**
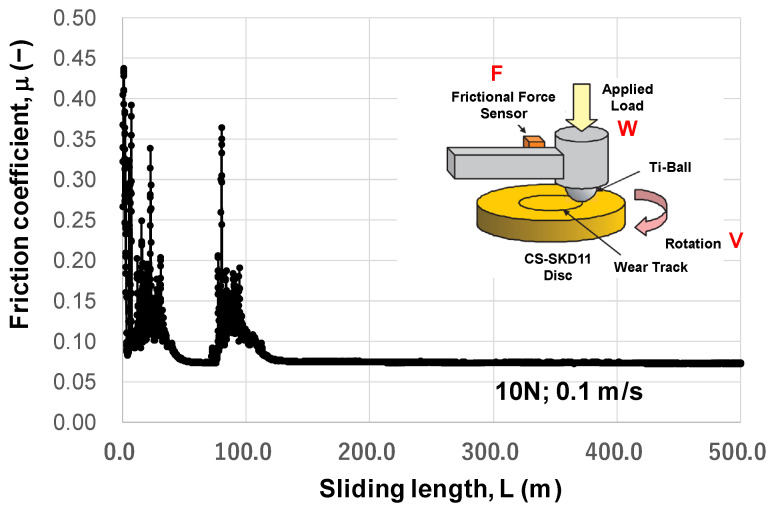
Variation in the measured friction coefficient with an increasing sliding distance under W = 10 N and v = 0.1 m/s.

**Figure 5:**
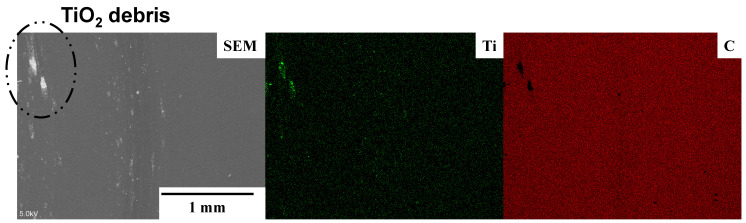
SEM image and titanium and carbon mapping analyzed by EDX on the wear track.

Variation in the measured friction coefficient with the sliding distance (L) is shown in Figure 4. Within L < 100 m, a spiky increase in the friction coefficient is detected, e.g., μ increases to 0.45 at L ~ 0 m and to 0.37 at L = 80 m. These metal sticking peaks prove that the titanium ball locally adhered to the MCSed SKD11 substrate. However, no sticking peaks are detected for L > 100 m; the friction coefficient remains constant at μ = 0.07. This stable frictional behavior proves that no adhesive wear occurs on the contact surface. This sliding distance of 100 m under W = 10 N and V = 0.1 ms is identified as an incubation time where the tribofilm is formed to cover the wear track on the MCSed SKD11 substrate through the nucleation and growth steps [18]. At L ~ 0 m, no tribofilm forms, so μ = 0.45 represents the friction coefficient of hardened SKD11 by MCS against the titanium ball. The gradual decrease in spiky peaks to μ = 0.07, reveals that the tribofilm nucleates and grows locally onto the wear track. Hence, this in situ formation of tribofilm requires the incubation time for full coverage of the contact surface by itself. The contact surface conditions at the wear track were analyzed by SEM-EDX after BOD testing for L = 500 m. As stated in [14], Figure 5 proves that no adhesives are detected at the wear track except for very few deposits of titanium oxide debris at the edge of the wear track. Most of the wear track is covered by the free-carbon film; this tribofilm reduces and preserves a low frictional state at μ = 0.07.

The stable friction coefficient, μ = 0.07, in Figure 4 is further discussed as follows. In [19,20], the POD (pin-on-Disc) and BOD testing results reported that μ >> 1.0, or seizure, occurs at the system of TiN and TiCN coatings against a pure titanium ball, and that μ > 1.0, or severe metallic sticking, is observed at a TiAlN coating against pure titanium. Those coatings suffer from severe galling of titanium debris onto the coating surface. In [21,22], 0.3 < μ < 0.5, or metal-sticking, happens repeatedly at a Si-bearing DLC coating against Ti, and μ ~ 0.2, or titanium debris, is locally detected at a nano-laminated DLC coating against Ti. These DLC coatings also suffer from the local adhesion of titanium fragments onto the coating surfaces. Hence, the low frictional state with μ = 0.07 in Figure 4 is attributed to the in situ formation of carbon tribofilm onto the contact surface of the MCSed SKD11 die to titanium counter material to prevent the die surface from direct contact with titanium.

### 3.2. In Situ Lubrication during Upsetting

**Figure 6:**
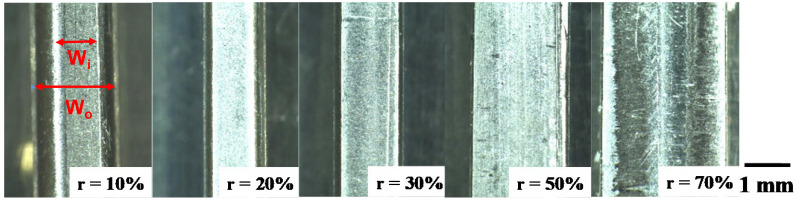
Variation in the upset titanium wires at r = 10%, 20%, 30%, 50%, and 70%.

**Figure 7:**
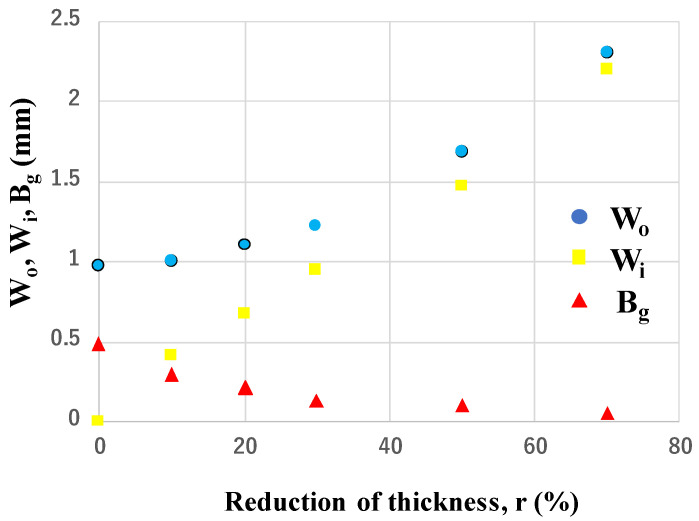
Variation in Wo, W_i_, and B_g_ with increasing thickness reduction (r) in upsetting.

The pure titanium bar with a diameter of 1.0 mm was upset in the laboratory-scale forging up to the specified thickness reduction (r) until r = 70%. Figure 6 shows the variation in the upset titanium work bars with increasing r. As stated in [14], a round wire deformed elasto-plastically by compression until r < 20%; the work flattened to a plate with homogeneous deformation for r > 50%. The upset wire width (W_o_), the contact surface width (W_i_), and the bulging displacement (B_g_), were measured at each specified thickness reduction, where B_g_ was defined by B_g_ = (W_o_ − W_i_)/2. As shown in Figure 7, W_o_ gradually increases with the thickness reduction from 1.0 mm to 2.35 mm, while W_i_ linearly increases from 0.0 mm at r = 0% and approaches W_o_ for r > 50%. Because of this increase in W_o_ and W_i_ with r, B_g_ monotonously decreases with r and B_g_ decreases to zero for r > 50%. 

**Figure 8:**
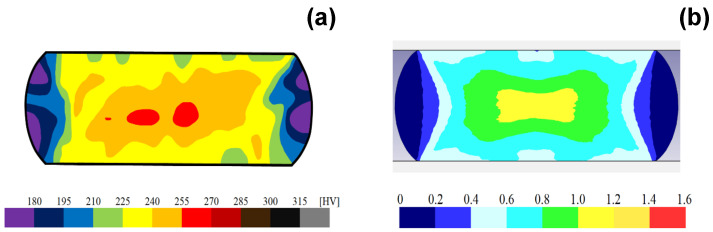
Comparison of the experimentally measured hardness map on the cross-section of the upset titanium wire at r = 50% to the calculated equivalent plastic strain distribution at r = 50%. (**a**) Hardness mapping at r = 50% and (**b**) the calculated equivalent plastic strain at r = 50%.

Following [23], this low frictional state was analyzed by the inverse analysis with the use of the finite element method. In its elasto-plastic analysis, the stress and strain distribution at each r were obtained by varying the friction coefficients parametrically. On the other hand, the hardness mapping was experimentally performed at each r. This hardness map at the cross-section of the wire corresponded to the equivalent plastic strain distribution. Then, μ at r = 50% was determined so that the experimentally obtained plastic strain map corresponded to the calculated equivalent plastic strain in the function of μ. Figure 8 compares the experimentally measured hardness map with the calculated equivalent plastic strain with μ = 0.05. A good agreement between the two distributions assures that μ = 0.05 at r = 50%. Following [24], the empirical estimate was performed using the relationship between the bulging displacement and μ. This method predicted that μ = 0.05 to 0.1. That is, the low frictional state on the MCSed die surface to the pure titanium work was validated empirically and theoretically.

Several studies have reported the frictional behavior at the contact surface between die and work under the upsetting process. When upsetting the aluminum alloy AA1060 by the WC (Co) die, it was found that μ = 0.22 at r = 50% in dry conditions, while μ = 0.07 at r = 50% under mist-lubrication [25]. Among the literature on the sliding test of titanium alloys on hardened steels, severe galling was commonly observed on the contact surface; i.e., μ >> 1.0 [26]. The homogeneous plastic flow of the titanium bar during upsetting, as shown in Figure 6 and Figure 7, reveals that the contact surface of MCSed SKD11 to the titanium work must be in situ-lubricated in a similar manner to the contact surface between the MCSed SKD11 substrate and the titanium ball in Figure 5. SEM-EDX analysis was performed to describe the contact surface condition after continuously upsetting the pure titanium work with r = 70%.

**Figure 9:**
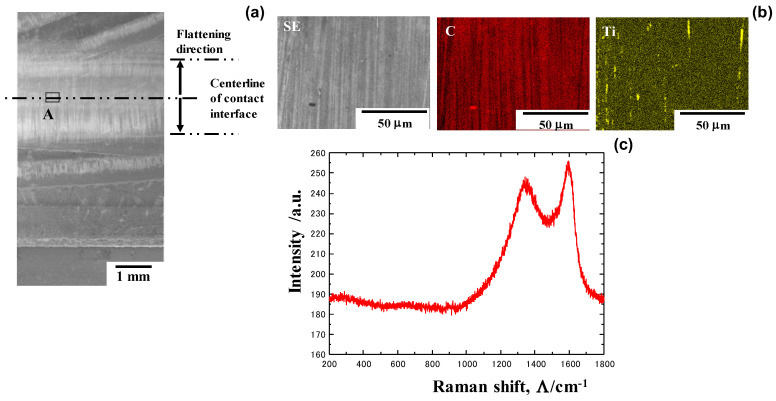
SEM-EDX and Raman spectroscopic analyses at the contact surface of the MCSed SKD11 die to the titanium work materials. (**a**) Low magnification SEM image on the contact surface, (**b**) SEM image and carbon and titanium maps at the A-region in (**a**), and (**c**) Raman spectrum measured at the A-region.

The SEM analysis with low magnification shown in Figure 9a reveals that a white stripe pattern is densely formed in the direction of metal flow on the contact surface. After SEM-EDX with a higher resolution, as shown in Figure 9b, this pattern corresponds to carbon stripes with a width of 1–3 μm. Titanium debris is scarcely detected on the contact surface. Remember that no carbon is detected on the MCSed SKD11 die surface because preliminary polishing and cleaning were performed before upsetting. These carbon stripes are in situ-formed only onto the contact surface of MCSed SKD11 to titanium bars. As reported in [26], Raman spectroscopy is sensitive enough to analyze the binding state of carbons in various carbon-base complexes. Figure 9c depicts the Raman spectrum at region-A in Figure 9a. Two main peaks are only detected at Λ = 1330 cm^−1^ and Λ = 1600 cm^−1^. This proves that free carbon without any binding states in carbides and carbon complexes is formed onto the contact surface, as stated in [27].

**Figure 10:**
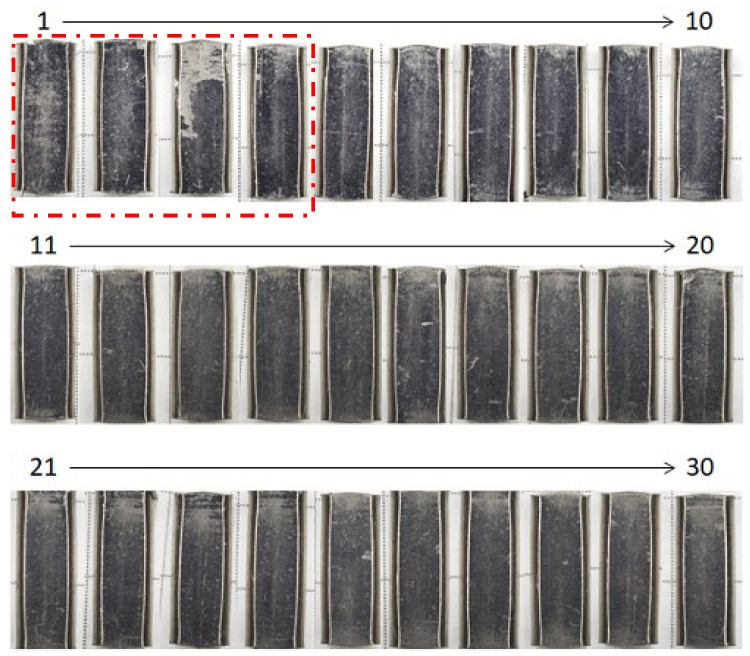
Variation in the upset titanium bars by r = 30% when increasing the number of strokes (N) from N = 1 to N = 30 in the case of the industry-scale upsetting process. The red one-dot chained line indicates the incubation time where the adhesion of work materials was detected.

The upsetting experiment at the industry scale was performed to demonstrate that the MCSed SKD11 die becomes free from severe galling in a similar manner to the laboratory-scale upsetting. As stated in [25], a size effect in friction testing and metal forming often disturbs the scientific effort to understand the fact. A pure titanium bar with a diameter of 5 mm was used as a feedstock for continuous upsetting by the knuckle-joint stamper. Figure 10 shows a series of upset works for r = 30% when increasing the number of strokes (N) from N = 1 to N = 30. As indicated by the enclosed one-dot chained line, the roughened surfaces were detected on the upset works at N = 1 to 4. No roughened surfaces were seen on the upset works for N > 5. Remembering that the initial MCSed SKD11 die surfaces were polished and cleaned before upsetting, this retardation of in situ lubrication during continuous upsetting corresponds to the incubation time during the BOD testing in Figure 5. After material science on the nucleation and growth mechanism [18], it required a duration for carbon solutes to isolate and diffuse from the inside of MCSed SKD11, to nucleate as a free-carbon agglomerate, and to grow to a free-carbon film on the contact surface. The incubation time in Figure 10 proves that the in situ lubrication by the free-carbon tribofilm works even in the industry-scaled forging process.

**Figure 11:**
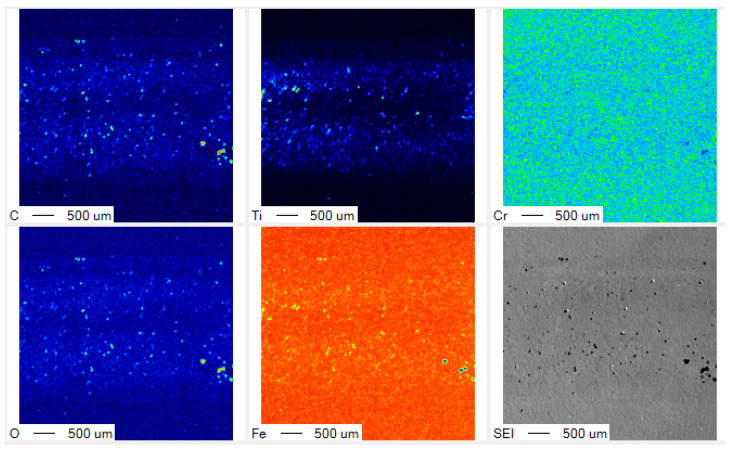
SEM-EDX analysis on the contact surface of the MCSed SKD11 die to work materials after N = 30 in the industry-scaled upsetting process. Brighter colors represent a higher content of each element detected by EDX.

SEM-EDX analysis was also employed to describe the contact surface condition after continuously upsetting by N = 30. Figure 11 depicts the SEM image and element mappings on the contact surface of the MCSed SKD11 after continuous upsetting for 30 strokes. No transfer of iron or chromium is detected on the contact surface. Although titanium oxide debris is slightly detected at the trace level, the whole interface is covered by the free-carbon film. That is, in Figure 10, the lack of a roughened surface on the upset titanium bar for N > 5 is attributed to in situ free-carbon tribofilm formation onto the contact surface of the MCSed SKD11 die to the titanium works. In the industry-scaled upsetting, the free-carbon tribofilm is in situ-formed on the contact surface to reduce friction and adhesive wear and not to roughen the work surfaces.

### 3.3. In Situ Lubrication during Near-Net Forging

**Figure 12:**
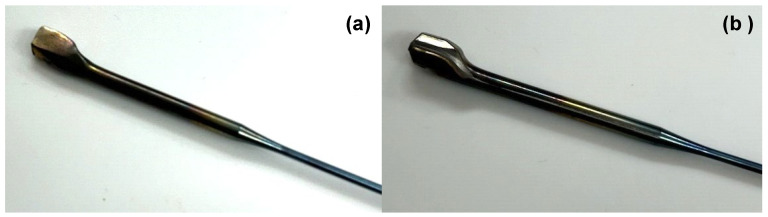
A near-net forging of the temple product from its preform in the final stage to fabricate the titanium eyeglass frame. (**a**) A temple preform before near-net forging and (**b**) a near-net forged temple.

**Figure 13:**
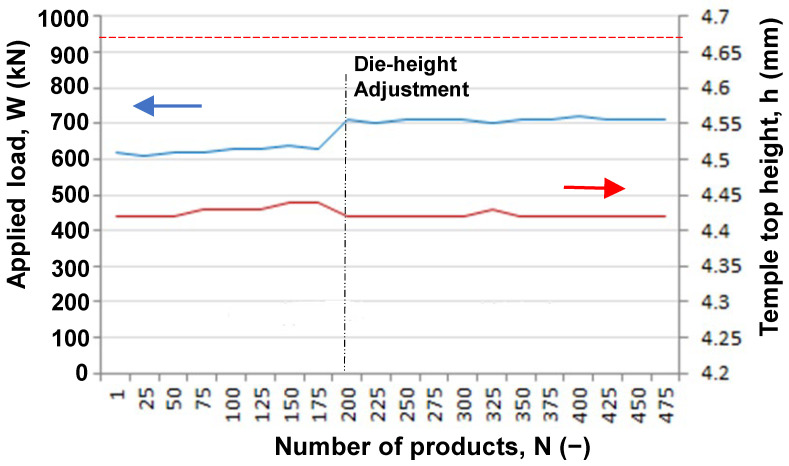
Variation in the applied load and temple top height when increasing the number of products. The average applied load using the bare ACD56 die is depicted by the red broken line.

The eyeglass frame consists of various middle-sized and tiny parts, which are made from round titanium bars. A temple is an essential part of supporting the eyeglass front frame with the designed decoration. In particular, dimensional accuracy and low surface roughness become a key item for this net-shaping of the temple. Figure 12 compares the temple preform and the forged temple by using the MCSed ACD56 dies. The temple top is shaped with sufficient accuracy by this forging. This height of the temple top (h) and the applied load (W) are employed as a process parameter to describe the near-net forgeability with the use of MCSed ACD56 dies. Figure 13 depicts the variation in W and h when increasing the number of products or the number of strokes, N. Although the die height was adjusted to suppress the height deviation at N = 200, both W and h became nearly constant by 700 kN and 4.42 mm, respectively, for N > 200. The applied load decreased by 30% from W = 965 kN when using bare ACD56 dies. This reduction in applied load reveals that the titanium work plastically deforms and flows with low friction and without galling onto the die surface in the presence of the free-carbon tribofilm. The temple top height (h) is preserved to be constant irrespective of N. In the conventional forging steps with the use of bare ACD56 dies, h gradually decreases or fluctuates with N. This also proves that a well-defined work flow in dry forging by using MCSed ACD56 dies results in the high surface quality of forged work.

**Figure 14:**
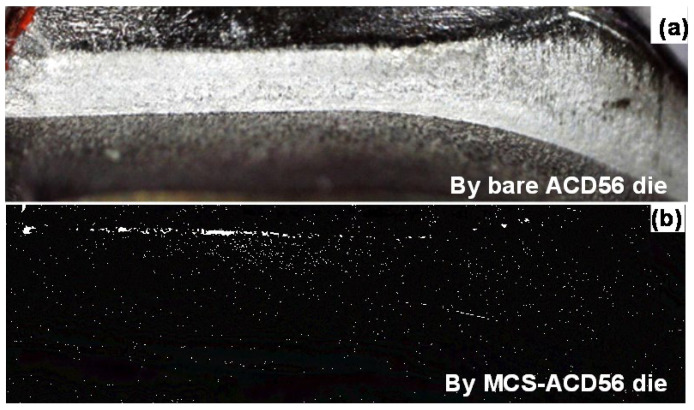
Comparison of the near-forged temple surface after upsetting with the use of ACD 56 dies with and without MCS treatment. (**a**) Forged temple surface using the bare ACD56 die and (**b**) forged temple surface using the MCSed ACD56 die.

Figure 14 compares the temple side surfaces after forging when using the bare ACD56 die and the MCSed ACD56 die. The surface roughing on the side surface of the work took place by local adhesion of fresh titanium materials onto the bare ACD56 die surface even in the early stage of the forging process. On the other hand, no visible roughness was seen on the surface of forged titanium even when N = 425. This significant improvement in the titanium side surface quality suggests that the pure titanium work deforms and flows in low friction without galling on the die surface and that the die surface condition is imprinted onto the work surface.

**Figure 15:**
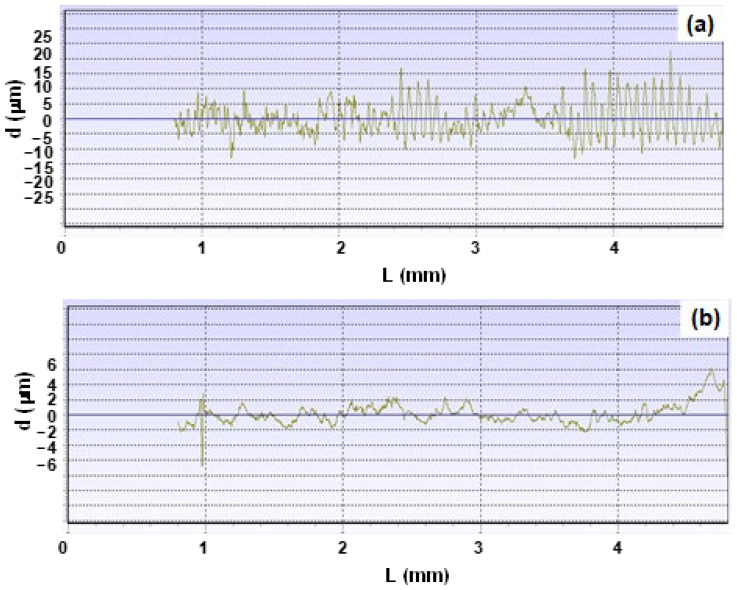
Comparison of the near-forged temple surface roughness profile after upsetting by using ACD 56 dies with and without MCS treatment. (**a**) Forged temple surface roughness profile using the bare ACD56 die and (**b**) forged temple surface roughness profile using the MCSed ACD56 die.

The surface roughness profile of the forged products using the ACD56 dies with and without MCS is also compared in Figure 15. Using the bare ACD56 die, the temple side surface roughness (d) significantly fluctuates around the average surface roughness with Ra = 4.12 μm. The peak-to-valley fluctuation intensity reaches 50 μm in Figure 15a. This reveals that the bumps of titanium work fragments adhere to the die surface during forging and the metal-sticking pattern imprints onto the temple side surface. On the other hand, the average surface roughness decreases from 4.12 μm to 0.99 μm by using the MCSed ACD56 dies. Most of the fluctuation intensity in roughing is suppressed within ±2 μm, much lower than the maximum roughness experienced when using the bare ACD56 dies. To be noticed, no steep valley-to-peak changes are seen in the measured surface profile in Figure 15b. This proves that the smooth die surface condition is imprinted onto the product surface to sustain the well-defined product surface conditions.

## 4. Discussion

Toward the SGDs (Sustainable Global Degrees), dry forging without using lubricating oils plays an essential role in significantly reducing the environmental burden and CO_2_ emissions from factories [28]. DLCs (Diamond-Like Coatings) and CVD–diamond coatings were utilized to reduce the amount of lubricating oils toward dry metal forming [29]. However, as reported in [30], every coating on the die materials suffered from the adhesion of titanium and titanium alloy debris and fragments during the dry stamping and forging processes. In addition, the solid lubricants never protected the die surfaces from the adhesion of their fresh works [4,30]. Solid lubrication via the in situ formation of the free-carbon tribofilm in Figure 1 only provides a solution to prevent the MCSed dies from the severe adhesion of titanium materials in dry forging. In this solid lubricating process, three stages play an important role to sustain this lubricating mechanism. 

In the first stage, the nano-structured layer is formed by the plastic straining process during plasma immersion carburizing into the SKD11 die, as explained in [31]. This layer has an amount of supersaturated carbon in the nano-sized grains and grain boundaries. These nano-grain boundary networks act as a carbon solute diffusion path. In the second stage, the isolated carbon solute in this nano-structured layer undergoes jumping diffusion through the nano-structured cluster boundaries to the hot spots on the contact surface under the stress gradient at the vicinity of the contact surface, as stated in [32]. In the third stage, these diffusing carbon solutes agglomerate themselves and grow to a film together with the plastic flow of the work during BOD testing, the upsetting, and the near-net forging, as shown in Figure 1. Hence, even when no carbons are present on the die surface before forging, the hot spots on the contact surface of the dies become black-colored with an increasing number of strokes, as stated in [5,14]. In the early stage of this formation mechanism of the free-carbon film, the growing film is not thick enough to lubricate the whole contact surface. After this incubation duration, the in situ-formed free-carbon film is preserved in the following forging steps. It should be noted that when the surfactants of die surfaces are mechanically or chemically removed, this incubation time must be shortened to start the in situ lubrication at the beginning.

Stainless steels and transient and refractory metals have been identified as difficult-to-form work materials, especially in dry conditions without lubricants [33]. DLC and CVD–diamond coatings have been frequently utilized in dry metal forming [34]. The measured friction coefficient was limited by 0.1 to 0.2. This reveals that the local adhesion of work materials must occur in the dry-forming steps. As stated in [35], the friction coefficients in dry forming were lowered to 0.05 or less under lubricating oils. No adhesive wear or galling took place on the lubricated contact surface of dies to work materials. This change of frictional behavior on the contact surface from dry to semi-dry or wet forming, is also true to the in situ solid lubricated state by the free-carbon tribofilm. As shown in the BOD testing and upsetting processes, a low frictional state with μ = 0.05 to 0.07 is sustained by the free-carbon tribofilm at all hot spots on the contact surface. The chemical inertness of the free-carbon film prevents the die surfaces from direct adhesion to work materials. As suggested by the molecular dynamics simulation in [4], once the die surface has a chemical interaction with the work materials, only in the form of potential between the two, galling occurs on the contact interface. That is, a low frictional state with μ < 0.1 without galling is only preserved by the chemical inertness of the free-carbon tribofilm on the contact surface.

The free-carbon tribofilm thickness is of much importance for the long-term usage of MCSed dies in practice. Based on the three-stage mechanism of in situ lubrication, this thickness is determined by the carbon content in the induced nanostructure at the vicinity of the die surface and by the applied stress gradient. In the former, since the average carbon content becomes nearly constant by 3 mass%, the carbon solute capacity for lubrication is estimated by the nanostructured layer thickness. In the latter, in situ lubrication is activated even by lowering W from 10 N to 5 N and by reducing V from 0.1 m/s to 0.05 m/s during BOD testing. The sensitivity of the carbon solute transportation process to the applied stress must be more important to estimate the limit of the stress gradient for the onset of carbon solute diffusion and agglomeration. Further study is necessary to investigate the effect of plasma carburizing conditions on the nanostructuring process and to study on the stress effect to induce carbon solute transportation along the nano-grain boundaries using SIMS (Secondary Ion Mass Spectroscopy) [36].

The little adhesive wearing state on the contact surface of MCSed dies to the work results in the low friction on the die surface and the low work hardening of work materials, as seen in Figure 6 and Figure 10. Furthermore, the applied load for forging is significantly reduced by 30% using the MCSed dies in Figure 13. In addition, this tribology in dry forging is free from the applied plastic strains in forging. In fact, as seen in Figure 7, a low frictional and wearing state is preserved even by varying the thickness reduction. Furthermore, as shown in Figure 13, it is also free from the number of strokes in near-net forging. Galling directly influences product quality. In case of the eyeglass frame, the metallic shining surface is valuable in market adaptivity to customers. As shown in Figure 14 and Figure 15, a rough surface significantly changes to be fine enough to be used as a semi-final product before polishing and finishing. In addition, this fine product surface quality is sustained throughout the forging process. In the conventional forging processes without MCS, the surface roughness in their early stage is enhanced to exceed the tolerance level in manufacturing. No die-maintenance and no intermediate modification of products are attractive to practical operation.

Titanium and titanium alloys have been utilized to fabricate various medical tools such as surgery knives, medical clips, artificial bones, and implants. Instead of the machining process, the present near-net forging process provides a method to shape near-net products through limited forging steps with low energy consumption and low labor cost. In particular, small- and medium-sized medical parts can be yielded from the bar feedstock in a series of dry forging processes to improve the cost-competitiveness and to save the raw materials.

## 5. Conclusions

Massively carbon-supersaturated dies are indispensable to making dry near-net forging of titanium and titanium alloy feedstocks with low friction, low wear, and high product quality. A low frictional and wearing state is sustained in the whole near-net forging processes by the in situ formation of free-carbon tribofilm at the hot spots on the contact surface between the MCSed die and work materials. This in situ lubrication mechanism works in laboratory- and industry-scaled forging processes. The well-defined tribology in near-net forging reflects the high quality of titanium and titanium alloy eyeglass frame parts. The high surface quality with much lower surface roughness improves the cost-competitiveness, saves additional treatment steps for finishing, and propels the production compatibility to eyeglass frame designs.

This in situ lubrication by the self-formation of the free-carbon tribofilm onto the die-to-work interface is applicable to dry or semidry forging of high-strength stainless steels and refractory metals with low friction and without galling. In particular, an alloying element design for forging dies might well be performed adaptive to this MCS process, e.g., in the case of tool steel die materials, Cr, Mo, or W contents must be effectively designed to drive MCS in the presence of these elements into the tool steel die. In addition, the plasma immersion carburizing process parameters must be effectively optimized to drive the three stages in this in situ solid lubrication mechanism. Furthermore, the MCSed tool steel punch and die life has to be surveyed through collaboration with industries for further applications in metal forming.

## Data Availability

The original contributions presented in this study are included in this article. Further inquiries can be directed to the corresponding author.
